# Genetic evaluation of migratory fish: Implications for conservation and stocking programs

**DOI:** 10.1002/ece3.6231

**Published:** 2020-09-16

**Authors:** Juliana da Silva Martins Pimentel, Sandra Ludwig, Leonardo Cardoso Resende, Pedro Ferreira Pinto Brandão‐Dias, Adriana Heloísa Pereira, Nazaré Lúcio de Abreu, Izinara Cruz Rosse, Ana Paula Vimieiro Martins, Susanne Facchin, João de Magalhães Lopes, Gilmar Bastos Santos, Carlos Bernado Mascarenhas Alves, Evanguedes Kalapothakis

**Affiliations:** ^1^ Department of Genetic, Ecology and Evolution Institute of Biological Sciences Federal University of Minas Gerais Belo Horizonte Brazil; ^2^ Pitágoras College Belo Horizonte Brazil; ^3^ Department of Zoology Institute of Biological Sciences Federal University of Minas Gerais Belo Horizonte Brazil; ^4^ Department of Pharmacy Federal University of Ouro Preto Ouro Preto Brazil; ^5^ Federal University of Lavras Lavras Brazil; ^6^ Pontifical Catholic University of Minas Gerais Belo Horizonte Brazil; ^7^ Bio‐Ambiental Consultoria Ltda Belo Horizonte Brazil

**Keywords:** fish stocking, genetic structure, genotyping, hydropower dam, next‐generation sequencing, São Francisco River

## Abstract

Fish stocking programs have been implemented to mitigate the blockage of original riverbeds by the construction of hydropower dams, which affects the natural migration of fish populations. However, this method raises concerns regarding the genetic rescue of the original populations of migratory fish species. We investigated the spatial distribution of genetic properties, such as genetic diversity, population structure, and gene flow (migration), of the Neotropical migratory fish *Prochilodus costatus* in the Três Marias dam in the São Francisco River basin, Brazil, and examined the possible effects of fish stocking programs on *P. costatus* populations in this region. In total, 1,017 specimens were sampled from 12 natural sites and a fish stocking program, and genotyped for high‐throughput sequencing at 8 microsatellite loci. The populations presented low genetic variability, with evidence of inbreeding and the presence of only four genetic pools; three pools were observed throughout the study region, and the fourth was exclusive to one area in the Paraopeba River. Additionally, we identified high unidirectional gene flow between regions, and a preferred migratory route between the Pará River and the upper portion of the São Francisco River. The fish stocking program succeeded in transposing the genetic pools from downstream to upstream of the Três Marias dam, but, regrettably, promoted genetic homogenization in the upper São Francisco River basin. Moreover, the data show the fragility of this species at the genetic level. This monitoring strategy could be a model for the development of conservation and management measures for migratory fish populations that are consumed by humans.

## Graphical abstract

We investigated the genetic diversity, population structure, and gene flow (migration) of the Neotropical migratory fish Prochilodus costatus in the Três Marias dam in São Francisco River basin, Brazil. The samples showed low genetic variability, with inbreeding and the presence of only four genetic pools. The fish stocking program succeeded in transposing the genetic pools from downstream to upstream of the Três Marias dam, but, regrettably, promoted genetic homogenization in the upper São Francisco River basin.

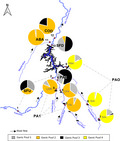

## 1 INTRODUCTION

Regarded as a “clean” energy source, hydropower contributes to 80% of the total share of global renewable energy (The World Bank, 2014). Hydropower dams in Brazil produce approximately 10% of the global hydropower (Agostinho, Gomes, Fernandez, & Suzuki, 2002; IEA, 2015; Sugunan, 1997; Zarfl, Lumsdon, Berlekamp, Tydecks, & Tockner, 2015). Despite their social and economic benefits to humans, hydropower dams block the natural flow of river courses, affecting downstream and upstream ecosystems. Successive dam construction impacts migratory fish populations (Agostinho et al., 2002); some reports suggest that dams are responsible for declines in migratory fish populations in the rivers of Brazil (Agostinho, Pelicice, & Gomes, 2008; Pelicice, Pompeu, & Agostinho, 2015; Pompeu, Agostinho, & Pelicice, 2012).

Studies in which population genetics tools are used to investigate gene flow and to identify migrants among the fish population are needed (e.g., Almada et al., 2017). Moreover, assessment of the genetic diversity status of fish species downstream and upstream of hydropower dams to detect possible effects of the dams and extensive fish stocking is an important step for conservation (Cooper et al., 2017; Savary et al., 2017; Vera‐Escalona, Senthivasan, Habit, & Ruzzante, 2018). In Brazil, the Companhia de Desenvolvimento Vale São Francisco (Codevasf) has implemented fish stocking programs at the Três Marias (TM) hydrobiology and fish farming station since 1980, with the release of thousands of fish broods into the upper São Francisco River. For example, about 100,000 fingerlings were released upstream of the TM dam into the upper São Francisco, Pará, and Paraopeba Rivers in 2002 (unpublished technical report, electrical power company of Minas Gerais, Brazil).

The São Francisco River, the fourth largest river in South America, is the longest river running entirely in Brazilian territory and an important source of hydropower, with six successive hydropower dams placed across its 2,900‐km extent. The upper portion of the São Francisco River basin is an important fishing site where multiple migratory species find favorable conditions for gonadal maturation and spawning (Sato, Bazzoli, Rizzo, Boschi, & Miranda, 2005). Since its construction in 1961, the TM hydropower dam has greatly affected natural migration and therefore the populations of migratory fish species, including *Prochilodus costatus* (Valenciennes 1850) (Agostinho et al., 2008; Lopes, Alves, Peressin, & Pompeu, 2018). This endemic fish species is a long‐distance migrator (Sato & Godinho, 2003), traveling up to 232 km (linear home range) during the reproductive season (Alves, 2007). *P. costatus* is an interesting study model because its downstream and upstream populations are separated by the TM dam, which impedes connection between the downstream and upstream river fragments, as it is not equipped with a translocation mechanism.

In this study, we evaluated the spatial distribution of genetic properties such as genetic diversity, population structure, and estimated gene flow (migration) of the migratory fish *P. costatus* from the upper São Francisco River basin. In addition, we investigated the possible effects of fish stocking programs on *P. costatus* populations in this region. This study is the first of its kind to examine a Neotropical migratory fish, with 1,017 individuals sampled in the main rivers of the region: Abaeté, São Francisco, Pará, and Paraopeba. Here, the term “fish stocking program” refers to the artificial translocation of downstream fish upstream of a dam (Sugunan, 1997). “Migration” refers to the natural migration of fish during the spawning season (Lucas & Baras, 2008). We used an innovative next‐generation sequencing (NGS) technique to genotype microsatellite sequences (simple sequence repeats), and genetic population approaches to characterize genetic homogenization and gene flow and to detect migration.

## 2 MATERIAL AND METHODS

### 2.1 Sampling and DNA extraction

**FIGURE 1:**
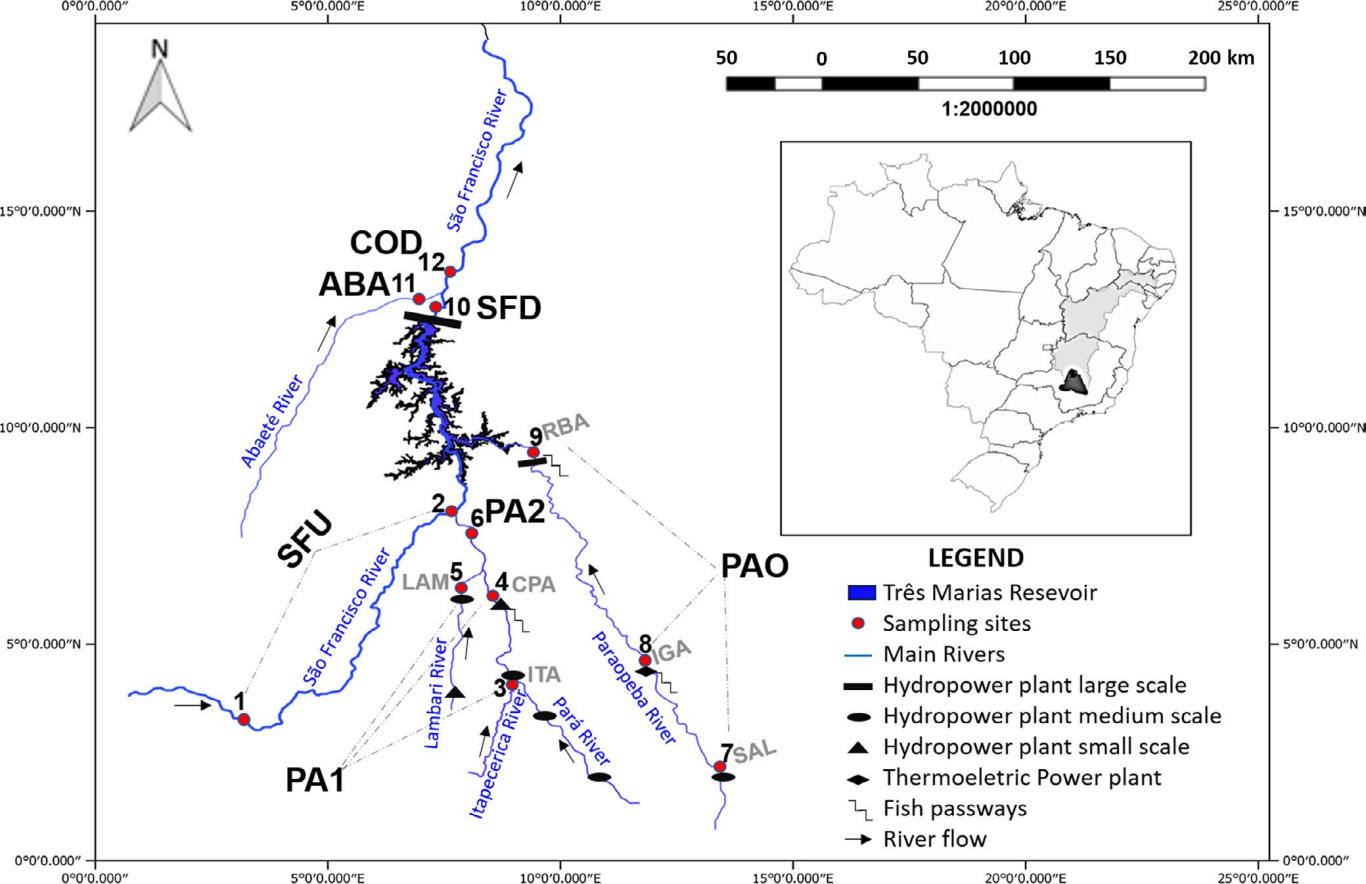
Upper São Francisco River basin, Brazil, showing the *Prochilodus costatus* sampling sites in the area of the Três Marias hydropower dam, including the sub‐basins of main tributaries (Pará and Paraopeba). Sampling sites (red circles): (1 and 2) São Francisco River upstream, (3) Itapecirica River, (4 and 6) Pará River, (5) Lambari River, (7–9) Paraopeba River, (10) São Francisco River downstream, (11) Abaeté River, and (12) Codevasf fish stocking site. More details on the sampling sites are provided in Table 1. This sampling area contains 16 hydroelectric undertakings and 1 thermoelectric undertaking. Only three undertakings have fish passways: a trap‐and‐truck fish translocation system at the Retiro Baixo hydroelectric power plant, and ladders at the Pitangui central hydraulic generator and Igarapé thermoelectric power plant

*Prochilodus costatus* sampling was carried out between 2013 and 2017 upstream and downstream of the TM hydropower dam in the upper São Francisco River basin (18°12′48″S, 45°15′40″ W; Table 1, Figure 1), at 12 sites distributed along four main rivers that were grouped by region: São Francisco River upstream (SFU; sites 1 and 2), Pará River section 1 (PA1; sites 3–5), Pará River section 2 (PA2; site 6), Paraopeba River (PAO; sites 7–9), São Francisco River downstream (SFD; site 10), Abaeté River (ABA; site 11), and the Codevasf fishing stocking facility (COD; site 12). The samples were grouped by region due to the lack of significant intraregional differences (*ϴ*st < 0.05). All individuals were captured using cast nets and gillnets, and small pieces of their caudal fins were cut and stored in 70% ethanol. All individuals were taxonomically identified based on Britski, Sato, and Rosa (1984) and then returned to the river. In the laboratory, fragments (12.5 mm^2^) of all caudal fin samples were placed into 96‐well plates to start the genomic DNA extraction protocol (detailed in Pimentel et al., 2018). Sampling was conducted as permitted by permanent collecting licenses issued by the Brazilian Instituto Chico Mendes de Conservação da Biodiversidade (protocol no. 57204‐1) and the Instituto Estadual de Florestas (protocol no. 014.007 2017).

**TABLE 1:** Sampling sites of *Prochilodus costatus* populations in the area surrounding the Três Marias hydropower dam, upper São Francisco River basin, Brazil

Sampling number	Region	Site	Coordinates	Regions	Sampling year	*N*
1	SFU	SFU	20°14′23.33″S 46°12′29.36″W	Samburá River—São Francisco River upstream^a^	2016/2017	36
2			19°12′30.30″S 45°9′39.52″W	São Francisco River upstream^a^	2014/2015	61
3	PA1	ITA	20°8′25″S 44°52′47″W	Itapecerica River	2014/2015/2016	89
4		CPA	19°43′01″S 44°54′11″W	Pará River—close to Conceição do Pará city	2014/2015/2016	46
5		LAM	19°36′51″S 45°06′26″W	Lambari River	2014/2015/2016	63
6	PA2	PA2	19°17′50″S 45°4′14.16″W	Pará River—close to São Francisco River upstream^a^	2014/2015/2016	96
7	PAO	SAL	20°30′24″S 43°59′12″W	Paraopeba River—close to Jaceaba city	2014/2015/2016	52
8		IGA	19°57′55″S 44°16′51″W	Paraopeba River—close to Juatuba city	2014/2015/2016	154
9		RBA	18°52′27″S 44°46′53″W	Paraopeba River—close to Felixlândia city	2014/2015/2016	79
10	SFD	SFD	18°11′35.39″S 45°15′14.29″W	São Francisco River downstream^a^	2014/2015/2016	236
11	ABA	ABA	18°2′15.60″S 45°11′48.77″W	Abaeté River	2013/2014	41
12	COD	COD	18°2′12.13″S 45°11′9.70″W	Codevasf fish stocking site downstream^a^	2013	64

^a^ Upstream and downstream in relation to the Três Marias dam.

### 2.2 Genotyping and treatment of data

The NGS approach was chosen in this study due to its high performance with large numbers of samples, low cost–benefit ratio, and rapid data acquisition. NGS genotyping and treatment of data were performed following Pimentel et al. (2018). Briefly, samples were genotyped using a pseudomultiplex strategy for eight microsatellite loci with tri‐ or tetra‐nucleotide repeats: ProC10, ProC18, ProC22, ProC36, ProC37, ProC44, ProC48, and ProC49 (Dryad Repository: https://doi.org/10.5061/dryad.tqjq2bvtq). All primers used contain overhang adapters compatible with the Illumina sequencing system employed and were incorporated into the target DNA through 30 polymerase chain reaction (PCR) cycles. An index primer containing the Illumina MiSeq adapter (which individualizes samples in NGS procedures) was attached to amplicons through 10 PCR amplification cycles, enabling locus identification for each individual using bioinformatics tools. Genotyping libraries were diluted using the Illumina buffer to attain the final concentration of 10 pMol DNA in a final volume of 1 ml. Sequencing was performed with 600 µl of the final solution using a nano kit (v. 2, 1M) and the MiSeq platform (Illumina^®^).

Raw reads with Phred < 30 and length < 75 pb were removed using Prinseq‐Lite (Schmieder & Edwards, 2011). To obtain correspondents to specific microsatellites, the filtered reads were mapped against reference sequences of the eight microsatellite loci (GenBank accession numbers Proc10 MG456705, Proc18 MG456707, Proc22 MG456708, Proc36 MG456709, Proc37 MG456710, Proc44 MG456712, Proc48 MG456715, and Proc49 MG456716) using the Bowtie2 software (Langmead & Steven, 2012), which generated mapping files in BAM format. After quality filtering, each obtained read was analyzed directly without the need to create contigs. The alignments obtained with Bowtie2 were analyzed using GATK (McKenna et al., 2010) to identify those most likely to be true alleles. This package requires the mapping files obtained with Bowtie2 and a variant call format (VCF) file that contains all possible variants (simple sequence repeats, single nucleotide polymorphisms, and indels) identified in a given region for the species under study. This VCF file was created using the SAMtools package (Li et al., 2009), which requires the reference sequences and Bowtie2 outputs to identify all possible variants of each individual at the eight loci. The resulting VCF file for each individual was concatenated in a single archive and provided as an input file for GATK (McKenna et al., 2010). The RealignTargetCreator and IndelRealigner tools of the GATK package were used to confirm the alignments obtained with Bowtie2 based on the variants obtained by SAMtools, with the elimination of low‐confidence reads from each Bowtie2 alignment.

The alleles at each locus were identified using the RepeatSeq software (Highnam et al., 2012) with the M2 parameter (the minimum mapping quality required, determined with Bowtie2 and confirmed with GATK). RepeatSeq inputs are the reference sequences, the validated alignment file obtained from GATK, and the.regions file that contains the location and motif repeat sequence out of eight loci. Thus, we created a.regions text file containing for each microsatellite the start and end positions of the repeat motif relative to the reference sequence, as well as the repeat motif sequence. Based on this information, RepeatSeq identified the motif repeat and defined the genotypes for each individual and locus according to the motif repeat length. For example, for an individual with the sequence 5′‐ATTATTATTATTATTATTATT‐3′ at locus Pcos10, the genotype in all mapped reads in this region is defined as 21/21, that is, homozygotic.

The GenotypeMicrosat.pl Perl script was developed to filter the genotyping results from RepeatSeq. We need to establish a minimum number of reads to infer the genotype. As individuals are diploids, only two alleles are possible and we establish whether they are homozygous or heterozygous according to the number of reads. Among the amplicons of a locus, if a given allele has more than 80% of reads, we consider that the individual is homozygous for that allele. If the allele with the largest number of reads does not exceed 80%, we consider the second allele, and if it encompasses ≥20% of the total reads, we consider the individual to be heterozygous. Rosa, Salvador, Bialetzki, & Santos, 2017; https://doi.org/10.1007/s00335‐016‐9670‐7) established a minimum of two reads to confirm the less frequent allele. In our work, we set the minimum depth at 10 reads, 20% of which would give at least two reads confirming the less frequent allele. This strategy was developed to avoid false positives. Loci that did not fulfill these criteria were identified as NA. The output of the script was a worksheet (in.xls format) containing the distribution of alleles per locus per individual.

### 2.3 Genetic diversity and population structure analyses

To evaluate the spatial genetic population structure for *P. costatus* populations in the upper São Francisco River basin, we estimated genetic indexes and pairwise *ϴ*st, and performed discriminant analysis of principal components (DAPC), comparing the eight study regions. The number of alleles (*A*), effective number of alleles (*Ae*), Shannon's index (I), observed heterozygosity (*Ho*), expected heterozygosity (*He*), and fixation index (*F*i) of *P. costatus* populations were analyzed using the GenALEx 6.5 software (Peakall & Smouse, 2006). The pairwise genetic population structure was evaluated using the *F* index through *ϴ*st (Weir & Cockerham, 1984), using GenALEx. The GenALEx software was also used to estimate whether loci were in Hardy–Weinberg equilibrium, and to identify linkage disequilibrium and determine the proportion of null alleles at each locus through the EM algorithm (Dempster, Laird, & Rubin, 1977), estimated using FreeNA (Chapuis & Estoup, 2006).

The DAPC, implemented with the *adegenet* R package (Jombart, 2008; Jombart, Devillard, & Balloux, 2010), was used to estimate diversity among groups of individuals. Through the *find.cluster* function, the data were transformed using principal component analysis. Next, the *k*‐means algorithm (*K* = number of clusters/groups of individuals characterized by allelic frequencies at each locus) was applied, and the proper genetic clusters were attributed using the Bayesian information criterion. Then, the membership probability of each individual for the different groups (*K*) was estimated using the *dapc* function based on the retained discriminant components. Two distinct combinations of the dataset were analyzed using the *compoplot* function: (a) an admixture model with correlated allele frequencies, which included *optim.a.score* information about the sampling regions to infer individuals with mixed genotypes from distinct populations; and (b) a possible hierarchical genetic structure separating regions. This multivariate analysis was implemented because it does not determine loci in Hardy–Weinberg equilibrium and does not require assumptions about the inheritance pattern of each locus (Dufresne, Stift, Vergilino, & Mable, 2014). These factors were important because we detected that the loci examined this study are not in Hardy–Weinberg equilibrium.

### 2.4 Migration and gene flow analysis and mixed genotypes

We used the *diveRsity* R package (Keenan, McGinnity, Cross, Crozier, & Prodöhl, 2013) with the *divMigrate* function and 0.35 filter threshold based on genetic indices to obtain estimates of migration and gene flow among the *P. costatus* populations sampled in the ABA, SFD, SFU, PA1, PA2, and PAO regions. We also estimated possible migration and gene flow for COD samples compared with those from the other regions to determine whether fish stocking was influencing the estimates. This method is known to detect asymmetric migration, that is, migration occurring at a significantly higher rate in one direction, according to the allele frequency data. The *D* (Crawford, 2010), *Nm* (Wright, 1943), and *G* (Nei, 1973) genetic indices were tested against our dataset.

The specimen with the highest probability of assignment from DAPC was considered the most likely source of the assigned genotype. Furthermore, individuals with mixed genotypes across the study populations were counted; all specimens identified at any site presenting more than one genetic pool were considered to have mixed genotypes.

## 3 RESULTS

### 3.1 Genetic diversity

From the 5,598,163 raw reads generated, 3,294,490 reads remained after quality trimming (https://datadryad.org/stash/dataset/doi:10.5061/dryad.tqjq2bvtq). On average, the genotyping produced 109.37 reads per population for Pcos10, 79.75 reads per population for Pcos18, 207.91 reads per population for Pcos22, 347.62 reads per population for Pcos36, 23.83 reads per population for Pcos37, 108.09 reads per population for Pcos44, 92.67 reads per population for Pcos48, and 700.97 reads per population for Pcos49. Only 3% of the produced genotypes for all individuals resulted in no allele amplification [due to nonamplification or low stipulation (<10 reads to support the existence of the allele)] and were designed as NA in the genotype. From the reads, we identified 63 alleles at the 8 loci analyzed for all *P. costatus*. *A* values ranged from 2 (ProC10 locus) to 12 (ProC49 locus), and the latter was the most polymorphic locus. Exclusive alleles were found in PAO (ProC48, allele 58, frequency 0.036), PA2 (ProC22, allele 39, frequency 0.031; allele 35, frequency 0.011; ProC44, allele 35, frequency 0.091), ABA (ProC49, allele 52, frequency 0.023), and SFD (ProC49, allele 47, frequency 0.014).

The genetic indices showed similar low genetic variability for the 12 sampled sites (*p* < .05). Overall, the *Ho* was low and significantly lower than the *He*, for all analyzed samples and loci. The lowest *Ho* was found for ABA and the highest was found for PA2 (Table 2). In addition, 77.5% of the analyzed loci indicated that the population was not in Hardy–Weinberg equilibrium, and the proportion of null alleles was not significant (Appendix S1.4). Furthermore, the *F*is ranged from 0.16 to 0.36 at the 12 sites, indicating inbreeding (Table 2).

**TABLE 2:** Genetic diversity indices (mean values) for *Prochilodus costatus* populations upstream and downstream of the Três Marias dam, São Francisco River, Brazil

Region	*N*	*A*	*Ae*	*I*	*Ho*	*He*	*Fi*
SFU	97	5	3	1.10	0.35	0.55	0.31
PAO	285	7	3	1.16	0.33	0.56	0.36
PA2	96	6	3	1.12	0.39	0.56	0.25
PA1	198	7	3	1.23	0.36	0.59	0.34
ABA	41	5	3	0.98	0.31	0.49	0.24
COD	64	5	3	0.98	0.37	0.49	0.16
SFD	236	7	3	1.18	0.35	0.58	0.33

Note

*N*, number sampled; *A*, number of different alleles; *Ae*, number of effective alleles; *I*, Shannon's index (diversity); *Ho*, observed heterozygosity; *He*, expected heterozygosity; *F*i, fixation index (inbreeding coefficient).

### 3.2 Genetic population structure

**FIGURE 2:**
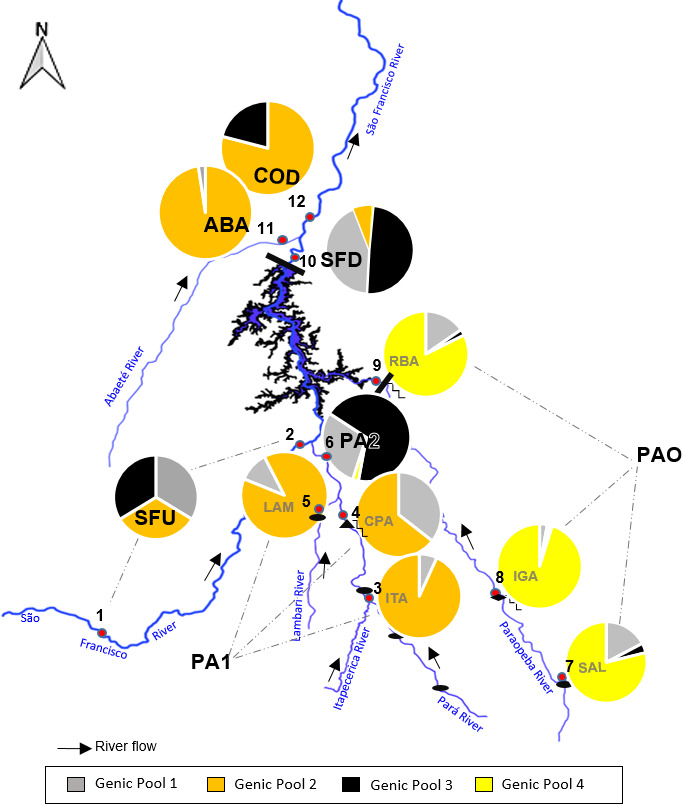
Gene pools and their frequencies found in *Prochilodus costatus* populations from the area of the Três Marias hydropower dam in the upper São Francisco River basin. The pie charts show the distributions of the gene pools and their frequencies in the sampled populations: (1 and 2) São Francisco River upstream (SFU), (3) Itapecerica River, (4 and 6) Pará River (PA1 and PA2), (5) Lambari River, (7–9) Paraopeba River (PAO), (10) São Francisco River downstream (SFD), (11) Abaeté River (ABA), and (12) Codevasf fish stocking site (COD)

Pairwise *θ*_ST_ analysis showed significant differences between some regions. Notably, the PAO values stood out in comparison with the other regions (Table 3). In the upstream regions, PA2 and PA1 presented moderate differentiation. In addition, a genetic structure was detected between PA2 (upstream of the TM dam) and the COD fish stock site and downstream ABA site. Pairwise comparison of the upstream regions PA1 and PAO with the downstream SFD also revealed a genetic structure. The SFD showed a genetic structure compared with the downstream ABA site and the COD fish stock site. Furthermore, DAPC identified four genetic pools distributed throughout the upper São Francisco River basin. Genetic pool 1 was shared among all populations except COD. Genetic pool 2 was the most frequent pool in the ABA (88%), COD (79%), and PA1 (85%) regions. Genetic pool 3 was the most frequent pool in the PA2 (69%) and SFD (49%) regions, and genetic pool 4 was found predominantly in the PAO region (86%; Figure 2).

**TABLE 3:** Pairwise *θ*_ST_ values for *Prochilodus costatus* populations collected in the area surrounding the Três Marias hydropower dam, upper São Francisco River basin, Brazil

	Regions	Upstream				Downstream	
		PA1	PA2	SFU	PAO	ABA	SFD
Upstream	PA2	0.1691*					
	SFU	0.0444	0.0574				
	PAO	0.1747*	0.1559*	0.1174*			
Downstream	ABA	0.000	0.1744*	0.0402	0.1733*		
	SFD	0.1481*	0.0128	0.0442	0.1425*	0.1496*	
	COD	0.0076	0.1539*	0.0322	0.1581*	0.0018	0.1309*

Note

The *θ*_ST_ range 0–0.05 indicates little genetic differentiation, 0.05–0.15 indicates moderate differentiation, 0.15–0.25 indicates pronounced differentiation, and >0.25 indicates very pronounced genetic differentiation (Wright, 1943).

Abbreviations: ABA, Abaeté River; COD, Codevasf fish stocking site; PA1 and PA2, Pará River; PAO, Paraopeba River; SFD, São Francisco River downstream; SFU, São Francisco River upstream.

* *p* < .005.

### 3.3 Migration, gene flow, and mixed genotypes

**FIGURE 3:**
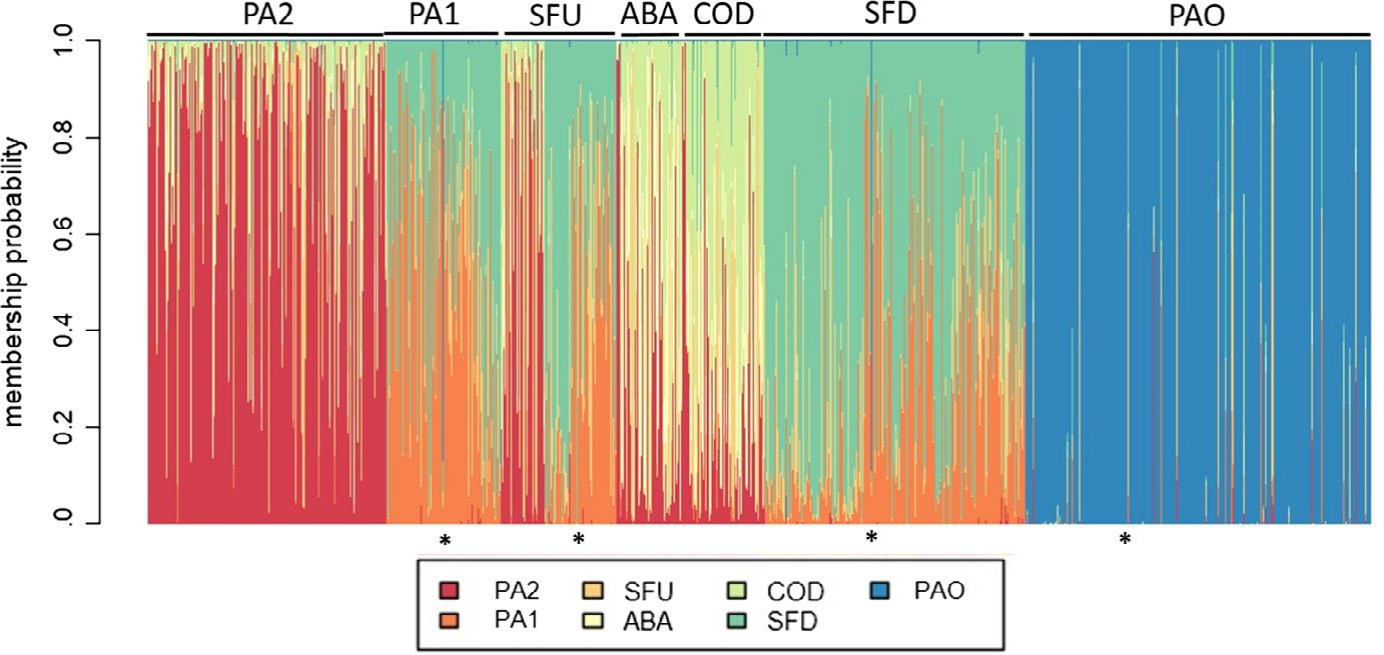
Membership probability of *Prochilodus costatus* individual assignments (columns) and probable population sources (indicated by distinct colors), based on the admixture pattern found in the area surrounding the Três Marias hydropower dam in the upper São Francisco River basin. The asterisks indicate examples of individuals with mixed genotypes (Appendix S1). PA2 and PA1, Pará River; SFU, São Francisco River upstream; ABA, Abaeté River; COD, Codevasf fish stocking site; SFD, São Francisco River downstream; PAO, Paraopeba River

DAPC analysis detected 256 individuals with mixed genotypes from distinct sites/regions, which were categorized as such because they had >50% probability of grouping with a different population (Figure 3). These individuals were probably early (September)/late (November) migrants (Appendix S1) or derived from a recent (same sampling year) previous generation, as juveniles take up to 2 years to reach reproductive maturity.

**FIGURE 4:**
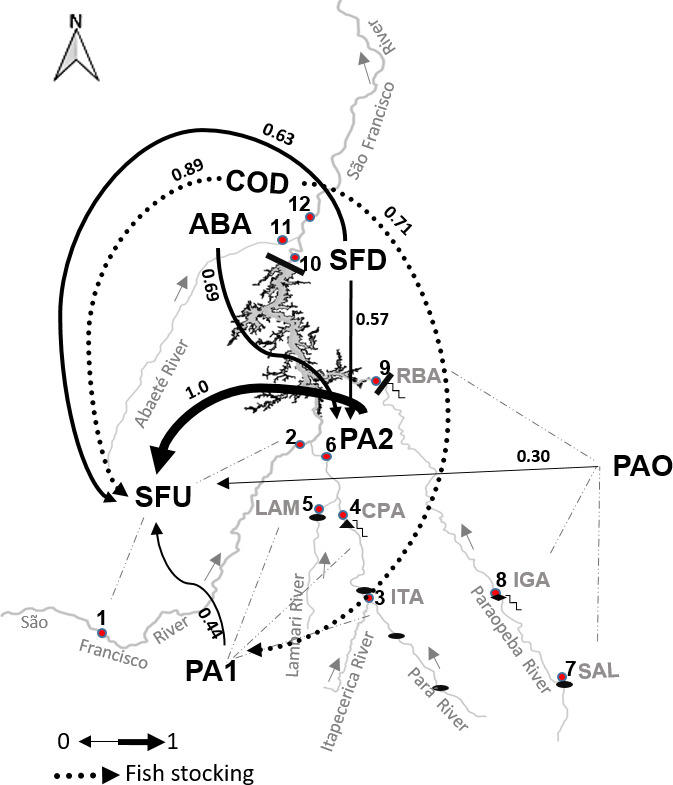
Migration and gene flow estimates, obtained with the *diveRsity* package, for the *Prochilodus costatus* populations sampled in the area surrounding the Três Marias hydropower dam. The arrows indicate the direction of gene flow and their thickness the strength of correlation between sites. The numbers adjacent to the arrows indicate the magnitude of gene flow (which increases with numerical value). COD, Codevasf fish stocking site (dotted arrows indicate the translocation by fish stocking); ABA, Abaeté River; SFD, São Francisco River downstream; PA1 and PA2, Pará River; SFU, São Francisco River upstream; PAO, Paraopeba River

The migration and gene flow estimates detected connectivity among all regions (*α* = .05). However, PAO showed low connectivity with the other study regions, despite the detection of few migrants. Furthermore, we identified unidirectional gene flow among the sampled regions, independently of the genetic index inferred (Figure 4). Based on the results, the detection of migrants between sampled sites corroborates the findings of the estimated gene flow analysis; however, these migrants did not likely contribute significantly to bidirectional gene flow, as the effective population sizes were low. We detected low gene flow (*p* < .5) between the PA1 and SFU regions, and moderate gene flow between the SFD and the SFU regions, as well as between the ABA site and the PA1 region. High gene flow (*p* > .7) was identified between the COD site and the PA1 region, and between the COD site and the SFU region. Notably, we detected high gene flow between the PA2 and SFU regions, suggesting the existence of intensive genetic exchange between these populations. The gene flow between the SFD and PA2 sites was moderate. However, all genetic diversity indices and migration rates indicated low connectivity between PAO and other regions. Indeed, migration analysis detected extremely low gene flow (*Nm* < 0.3) among sites in the SFU region.

## 4 DISCUSSION

In the present study, we investigated the spatial distribution of genetic diversity, gene flow, and migratory routes of *P. costatus* in the upper São Francisco River basin. We additionally evaluated the genetic impact of the Codevasf broodstock matrices by comparing the genetic pools of *P. costatus* populations from downstream and upstream of the dam. The NGS methodology employed herein allowed us to evaluate a large amount of samples and generate a high volume of genomic data; with the associated use of bioinformatics tools, we were able to improve resolution, confiability, and timing, as detailed in Pimentel et al. (2018).

The genomic data enabled us to obtain genetic population indices, such as those of heterozygosity and endogamy, for *P. costatus* populations in the upper São Francisco River basin, including the areas surrounding the TM dam. These indices were low, as reported in previous studies of *P. costatus* populations from the downstream region of the TM dam using similar microsatellite regions (tri‐ and tetra‐nucleotides) (Carvalho‐Costa, Hatanaka, & Galetti, 2008; Melo, 2011; Sanchez, Spina, & Perera, 2012). The significant deviations found in this study from Hardy–Weinberg equilibrium and the heterozygote deficit found at all loci may be explained by the presence of mating systems and population structures (i.e., the Wahlund effect). These findings may corroborate those of previous studies, which suggested the coexistence of different genetic populations of other rheophilic fishes and their comigration along the main river channel (Wasko & Galetti, 2002; Hatanaka & Galetti, 2003; Hatanaka, Henrique‐Silva, & Galetti, 2006).

Most Codevasf matrices were collected downstream of the TM dam. The distribution patterns of the four gene pools identified in the areas surrounding the TM dam suggest that the *P. costatus* populations in these areas are connected, but in a limited way, with those of the Paraopeba River. Gene pool 1 was occasionally identified in the populations of the Paraopeba River and was distributed widely between the TM dam downstream sites of the São Francisco and Pará rivers. Gene pool 3 was present at high frequencies in SFD, SFU, and PA2, and gene pool 2 was present at high frequencies in the ABA, COD and PA1 sites upstream of the TM dam, suggesting the occurrence of gene flow among these *P. costatus* populations. As the passage of fish to upstream of the TM dam is unlikely (Pelicice et al., 2015), the fish stocking programs conducted by Codevasf may have contributed to this genetic connection. Codevasf has been stocking fish in the region surrounding the TM dam for more than 20 years, with reproductive matrices collected downstream of the TM dam (Codevasf unpublished data). Surprisingly, fish sampled at the Paraopeba River site were the only ones with gene pool 4, which was present at high frequency (>90%). Environmental factors can trigger spawning seasons that affect recruitment area selection (Rosa et al., 2017), and likely have changed allele frequencies at the population level (see more details in Appendix S2).

The *P. costatus* population of the Paraopeba River also differs from the populations at other sampling sites in the low frequencies of gene pools 1 and 3, and the presence of only four SFU and PA1 migrants, suggesting that gene flow occurred only in the past or that current gene flow is very limited. One possible explanation is the existence of the TM dam, which may represent the greatest physical barrier to the natural migration of fish (Lopes et al., 2018). Another possibility is that the genetic profile of the population sampled in PAO was also influenced by the lack of Codevasf fish stocking activities. In 2002, Codevasf fish stocking involved the release of few (~16,000) fingerlings into the Paraopeba River compared with those released at PA2 (~50,000). Most importantly, stocking has been interrupted since that time (CEMIG unpublished data).

Taken together, our data suggest that Codevasf's fish stocking procedures, performed from downstream to upstream of the TM dam, and transposed the ABA gene pools to upstream sites, such as SFU and PA1. On the other hand, genetic data from the 2013 reproductive matrices of the fish culture station maintained by Codevasf suggest inbreeding. The *F*is and *A* for the *P. costatus* populations in the study regions were similar to those obtained in previous studies (Carvalho‐Costa et al., 2008; Melo, Sato, Foresti, & Oliveira, 2013) and to those of other Neotropical migratory fishes (Sanchez et al., 2012. Several studies of repopulation with native salmonids in various parts of the world have shown that stocking has little impact on the genetic makeups of populations (e.g., Allendorf & Seeb, 2000; Eldridge & Killebrew, 2008; Gow, Tamkee, Heggenes, Wilson, & Taylor, 2011; Heggenes, Beere, Tamkee, & Taylor, 2006; Small, Currens, Johnson, Frye, & VonBargen, 2009; Stelkens, Schmid, Selz, & Seehausen, 2009). In contrast, other works have suggested that intense fish stocking can lead to increased genetic diversity (e.g., Ferreira et al., 2017; Marie, Bernatchez, & Garant, 2010; Valiquette, Perrier, Thibault, & Bernatchez, 2014) and the introduction of genes, thereby effecting the genetic rescue of endangered populations by alleviating inbreeding depression and boosting fitness (Ingvarsson, 2001; Tallmon, Luikart, & Waples, 2004).

In addition to the intrinsic characteristics of migratory species that favor high genetic diversity, other factors, such as connectivity between areas and the storage process, may directly influence genetic diversity (Ferreira et al., 2017). For example, *Prochilodus* sp*.* can migrate up to 237 km (Alves, 2007) and may recruit individuals from different regions to complete the reproductive process. Thus, we suggest that the matrices used to repopulate the region under the influence of the TM dam be further investigated in the search for the one most likely to guarantee greater genetic diversity of this important migratory fish.

Genetic studies of migratory fish species, especially those consumed by humans, are extremely important in identifying the fragility of these species and helping to develop conservation measures to reduce the human impacts on these populations (Eldridge & Killebrew, 2008; Heggenes et al., 2006; Prado et al., 2017). The identification of priority sites and proper means of their environmental protection may help to maintain the genetic diversity of migratory fish populations. Our results suggest that conservation sites should include the SFU region, which has three of the four sets of gene pools detected for *P. costatus*, and PAO, which has a unique genetic profile that probably represents an adaptation of *P. costatus* to this region.

The large number of genetic samples evaluated in this study allowed the prediction of natural migration routes adopted by populations of *P. costatus* in regions surrounding the TM dam, in addition to the identification of prospective priority preservation sites. Thus, we would like to emphasize the importance of considering genetic criteria in selecting reproductive matrices of fish, to avoid genetic homogenization and loss of important genotypes for the population. Recent studies involving landscape genetics have sought to understand how interactions between landscape features and evolutionary processes, such as gene flow, genetic drift, and selection, interfere with the spatial distribution of genetic variation (Cushman, McKelvey, Hayden, & Schwartz, 2006; Holderegger & Wagner, 2006; Lowry, 2010; Manel, Schwartz, Luikart, & Taberlet, 2003; Storfer, Murphy, Spear, Holderegger, & Waits, 2010). Future studies in Neotropical systems, including those employing landscape genetics, could expand our knowledge about fish populations and may help to further the development of wildlife management and conservation strategies in regions strongly impacted by human activities.

## CONFLICT OF INTEREST

The authors declare that the present research was conducted in the absence of any commercial relationship and that they have no conflict of interest.

## AUTHOR CONTRIBUTION

**Juliana da S. M. Pimentel:** Conceptualization (lead); data curation (equal); formal analysis (lead); funding acquisition (supporting); Investigation (lead); Methodology (lead); project administration (equal); resources (equal); software (supporting); supervision (equal); validation (lead); visualization (lead); writing – original draft (lead); writing – review and editing (lead). **Sandra Ludwig:** Conceptualization (equal); data curation (lead); formal analysis (lead); funding acquisition (supporting); investigation (lead); methodology (equal); project administration (supporting); resources (supporting); software (lead); supervision (equal); validation (lead); visualization (lead); writing – original draft (lead); writing – review and editing (lead). **Leonardo C. Resende:** Conceptualization (equal); data curation (equal); formal analysis (equal); investigation (equal); methodology (equal); resources (equal); validation (equal); visualization (equal); writing – original draft (equal); writing – review and editing (equal). **Pedro F. P. Brandão‐Dias:** Conceptualization (supporting); formal analysis (supporting); investigation (equal); methodology (equal); resources (supporting); validation (supporting); writing – original draft (supporting); writing – review and editing (supporting). **Adriana H. Pereira:** Conceptualization (supporting); formal analysis (supporting); investigation (supporting); methodology (supporting); software (supporting); writing – original draft (supporting). **Nazaré L. de Abreu:** Data curation (supporting); formal analysis (supporting); methodology (supporting). **Izinara C. Rosse:** Formal analysis (supporting); methodology (supporting); software (lead); validation (equal); writing – original draft (supporting); writing – review and editing (supporting). **Ana P. V. Martins:** Formal analysis (supporting); funding acquisition (supporting); software (supporting); writing – original draft (supporting). **Susanne Facchin:** Data curation (supporting); formal analysis (supporting); methodology (supporting). **João de M. Lopes:** Data curation (supporting); funding acquisition (supporting); investigation (supporting); project administration (supporting). **Gilmar B. Santos:** Data curation (supporting); funding acquisition (supporting); project administration (supporting). **Carlos B. M. Alves:** Data curation (supporting); investigation (supporting). **Evanguedes Kalapothakis:** Conceptualization (equal); data curation (equal); formal analysis (supporting); funding acquisition (lead); investigation (equal); methodology (equal); project administration (lead); resources (equal); software (supporting); supervision (lead); validation (equal); visualization (equal); writing – original draft (equal); writing – review and editing (equal).

## Supporting information

Appendix S1

Appendix S2

## Data Availability

Archived microsatellite data are available in the Dryad repository (https://doi.org/10.5061/dryad.tqjq2bvtq) and can be accessed at the temporary link https://datadryad.org/stash/share/xovKEPenZcKgUgAAL4aLtsIRdR_4KphvAvYlCkG‐i_c . Raw sequences from the genotyping are available in GenBank under the NCBI BioProject accession number PRJNA548358.
