# Supplementary material for: Genetic evaluation of migratory fish: Implications for conservation and stocking programs
Source: Ecol Evol. 2020 Sep 16;10(19):10314–24. doi: 10.1002/ece3.6231 (PMC7548202; doi:10.1002/ece3.6231)

**Appendix S1**

1. DAPC


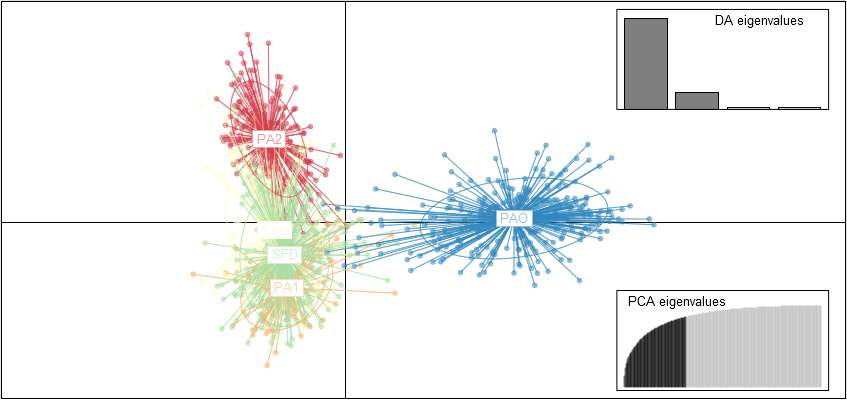


1. HWE for microsatellite loci by regions:


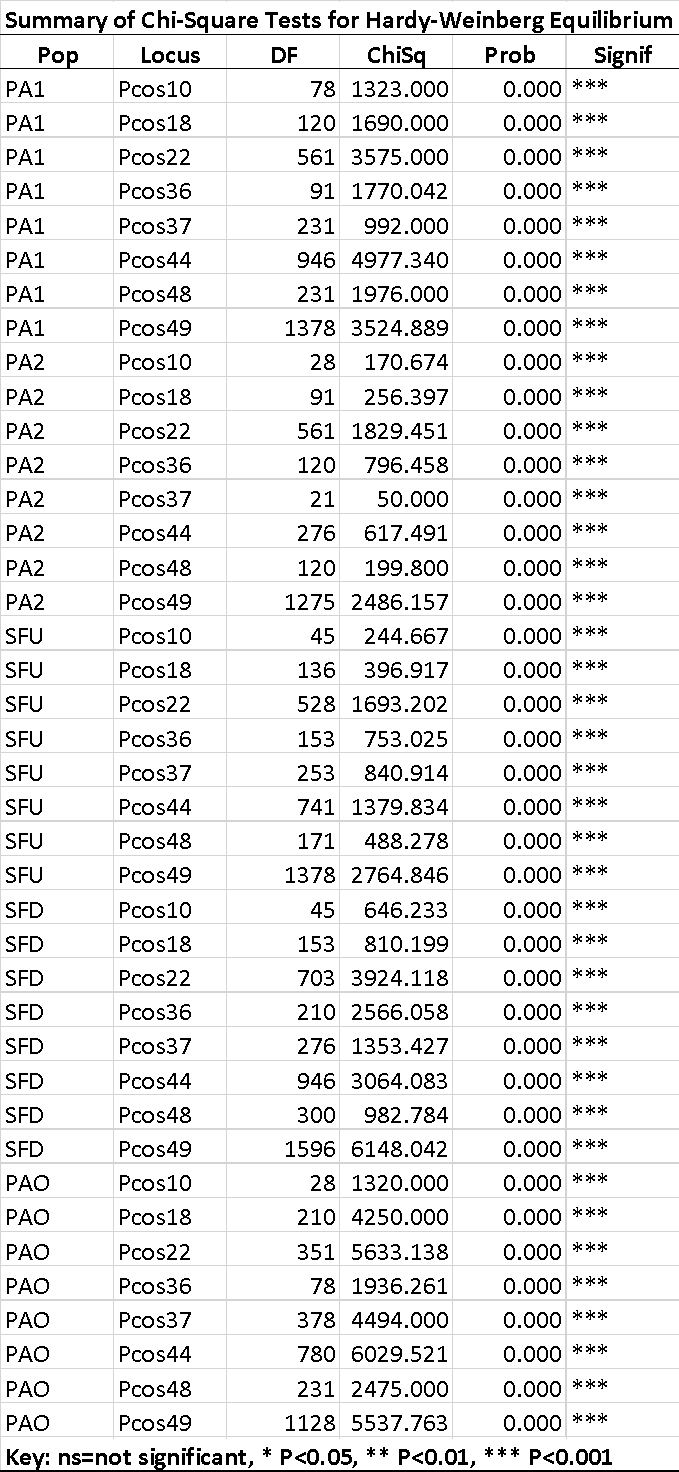


1. Table of migrants of *Prochilodus costat*us detected for São Francisco River.

| Migrants of *Prochilodus costatus* detected through discriminant analysis of principal components, sampling regions and plausible population sources. | | | | | | | | | |
| --- | --- | --- | --- | --- | --- | --- | --- | --- | --- |
| **Sampling region** | ***N*** | **Population source** | | | | | | | **Number of migrants** |
|  |  | Upstream | | | | Downstream | | |  |
|  |  | PA2 | PA1 | SFU | PAO | ABA | SFD | COD |  |
| Upstream |  |  |  |  |  |  |  |  |  |
| PA2 | 96 |  | 22 | 3 | 2 |  | 41 |  | 68 |
| PA1 | 198 |  |  | 8 |  | 5 | 2 | 11 | 26 |
| SFU | 97 | 11 | 23 |  |  | 1 | 35 | 10 | 80 |
| PAO | 285 | 2 |  | 9 |  |  | 2 |  | 11 |
| Downstream |  |  |  |  |  |  |  |  |  |
| ABA | 41 |  | 13 |  |  |  |  | 8 | 21 |
| SFD | 236 | 30 |  |  | 2 |  |  |  | 32 |
| COD | 64 |  | 8 | 6 |  | 2 | 2 |  | 18 |
| ← Migration direction | | | | | | | | | |

*N*, number sampled; PA1 and PA2, Pará River; SFU, São Francisco River upstream; PAO, Paraopeba River; ABA, Abaeté River; SFD, São Francisco River downstream; COD, Codevasf fish stocking site.

1. Results of linked disequilibrium and the proportion of null alleles of each locus.


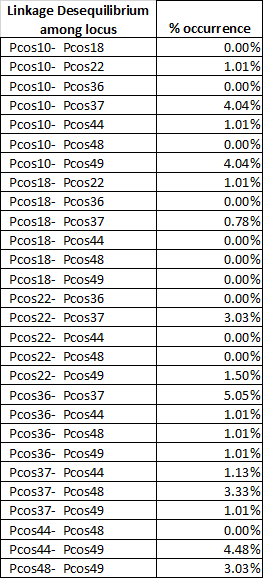


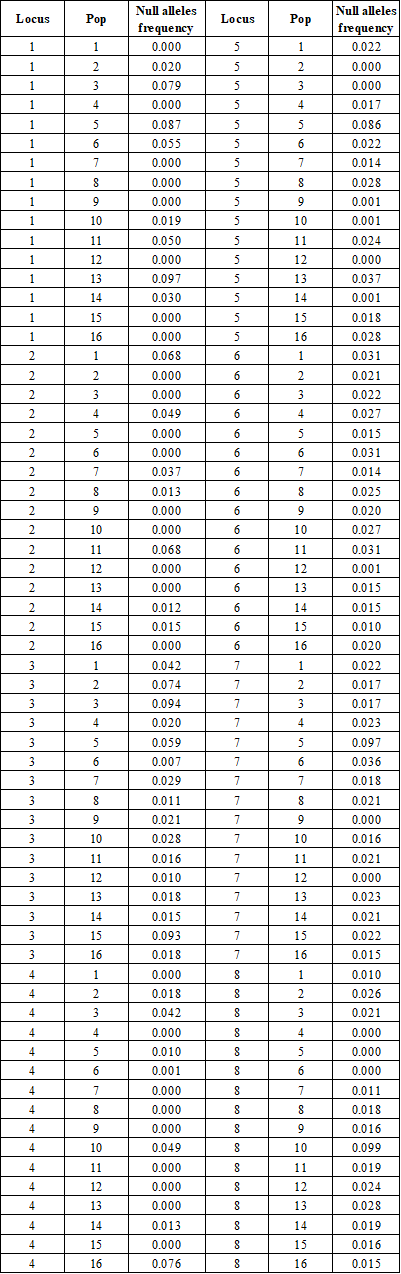

Supplement: Supplementary file 1 — Appendix S1 [file ECE3-10-10314-s001.docx]
