# Supplementary material for: Genetic evaluation of migratory fish: Implications for conservation and stocking programs
Source: Ecol Evol. 2020 Sep 16;10(19):10314–24. doi: 10.1002/ece3.6231 (PMC7548202; doi:10.1002/ece3.6231)
Supplement: Supplementary file 2 — Appendix S2 [file ECE3-10-10314-s002.docx]

**Appendix S2**

Discussion Details

Most Codevasf matrices were collected downstream of the TM dam. The distribution patterns of the four gene pools identified in the areas surrounding the TM dam suggest that the *P. costatus* populations in these areas are connected, but in a limited way, with those of the Paraopeba River. Gene pool 1 was occasionally identified in the populations of the Paraopeba River, and was distributed widely between the TM dam downstream sites of the São Francisco and Pará rivers. Gene pool 3 was present at high frequencies in SFD, SFU, and PA2, suggesting the occurrence of gene flow among these *P. costatus* populations. As the passage of fish to upstream of the TM dam is unlikely (Pelicice et al. 2015), the fish stocking programs conducted by Codevasf may have contributed to this genetic connection, as reflected by the distribution pattern of gene pool 2. Indeed, this pool was present at high frequencies at the ABA, COD (in the 2013 Codevasf reproductive matrices), and PA1 sites upstream of the TM dam. Genetic structuring data obtained for these populations corroborate their proximity. As mentioned previously, Codevasf has been stocking fish in the region surrounding the TM dam for more than 20 years, with reproductive matrices collected downstream of the TM dam (Codevasf unpublished data). The 64 individuals from the fish stocking program used in this study represented the broodstock used by Codevasf in 2013 and provided data on the genetic aspects of the populations downstream of the TM dam and on the effect of the stocking program carried out upstream of the dam.

Our results show a preferential natural migration route between PA2 and SFU (Nm = 1.0), as these sites shared two gene pools (1 and 3) at high frequencies and showed a low degree of genetic difference (*θ*st = 0.0574). Using radiotelemetry, Lopes et al. (2018) found that *P. costatus* populations preferentially use this natural migratory route. The high gene flow between PA2 and SFU is probably due to the free traffic between the São Francisco and Pará rivers, and especially to the lack of physical barriers blocking movement during the natural migration and reproductive periods. In addition, the São Francisco River is large, with more water flow than the Pará River, and thus may be more attractive for reproductive migration. On the other hand, the estimated gene flow between SFD and PA2 could only have been promoted artificially by Codevasf (through the fish stocking program), which also explains the sharing of high frequencies of genetic pools 1 and 3.

Interestingly, although the Pará River regions (PA1 and PA2) shared gene pool 1, they harbored two different populations (*θ*st = 0.1641). Indeed, samples from the PA1 region showed a high frequency of gene pool 2 and the absence of gene pool 3. According to Rosa et al. (2017), spawning sites in the PA1 region support the existence of a natural migratory route for individuals in this region. Our results suggest that PA1 fish migrate and reproduce in this area, whereas PA2 fish migrate preferentially to the São Francisco River.

Previous studies have shown that changes in hydrological pattern, i.e., in water flow or quality, caused by the installation of dams affect the natural migration and spawning of rheophilic species (Humphries and Lakes 2000; Agostinho et al. 2004; Bailly et al. 2008). The Pará River has several small and medium-sized barriers that may be altering the downstream flow peaks and water quality parameters, such as turbidity. Thus, the DAPC and gene flow results suggest that the dams constructed along the Pará River could discourage natural migration of the populations in the region (i.e., from PA2 to PA1, and to SFU), probably by suppressing the environmental stimuli that trigger migration (Lopes et al. 2018).

Surprisingly, fish sampled at the Paraopeba River site were the only ones with gene pool 4, which was present at high frequency (>90%). Moreover, the genetic structure differed significantly (*θ*st > 0.11) from those of the other sites analyzed in this study. The specific genetic profile found in the PAO samples may be the result of a recent population bottleneck (data not shown). Population structure in fish has been justified by the “bottleneck effect” (Pil et al. 2017). These authors suggested that paleoclimatic changes are responsible for such events. However, we hypothesized that the particular environmental characteristics of the Paraopeba River have contributed to the genetic differences observed. The Paraopeba River basin is greatly affected by human activities, and the water quality index close to the sampling sites of the current study has been considered to be poor or very poor (IGAM 2013). This river has been receiving industrial and mining waste for more than a century, and Veado et al. (2000) reported increased concentrations of polluting metals in an area of industrial discharge. Environmental factors can trigger spawning seasons that affect recruitment area selection (Rosa et al., 2017), and likely have changed allele frequencies at the population level. Such interference has also been observed in populations of salmon (*Salmo salar* [Linnaeus](https://en.wikipedia.org/wiki/Carl_Linnaeus), [1758](https://en.wikipedia.org/wiki/10th_edition_of_Systema_Naturae)) (Ozerov et al. 2012), which reveals that environmental characteristics are associated strongly with genetic differences.

**Reference**

Agostinho AA, Gomes LC., Veríssimo S, Okada EK (2004). Flood regime, dam regulation and fish in the Upper Paraná River: effects on assemblage attributes, reproduction and recruitment. *Rev. Fish. Biol. Fish.*, 14: 11-19.

Bailly D, Agostinho A, Suzuki H I (2008). Influence of the flood regime on the reproduction of fish species with different reproductive strategies in the Cuiaba River, upper Pantanal, Brazil. *River Res. Appl.*, 24, 1218–1229.

Humphries P, Lakes PS (2000). Fish larvae and the management of regulated rivers. *Regul. Rivers: Res. Manage.* Chichester, 16(1/2): 421-432.

IGAM - Instituto Mineiro de Gestão das Águas. (2013). *Identificação de municípios com condição crítica para a qualidade de água na bacia do rio Paraopeba*. Belo Horizonte: IGAM, 41p.

Lopes JM, Alves CBM, Peressin A, Pompeu P (2018). Influence of rainfall, hydrological fluctuations, and lunar phase on spawning migration timing of the Neotropical fish *Prochilodus costatus.* *Hydrobiologia*: 1-17. https://doi.org/10.1007/s10750-018-3601-4

Ozerov, M. Y., Veselov, A. E., Lumme, J., Primmer, C. R. (2012). “Riverscape” genetics: river characteristics influence the genetic structure and diversity of anadromous and freshwater Atlantic salmon (*Salmo salar*)

Veado MAR, de Oliveira AH, Veado, J. C. C., Revel G, Pinte G (2000). Analysis and distribution of metals in the Paraopeba and the Das Velhas Rivers, Brazil. *Water SA*, *26*(2), 249-254.

Pelicice FM, Pompeu PS, Agostinho AA (2015). Large reservoirs as ecological barriers to downstream movements of Neotropical migratory fish. *Fish Fish.*, 16(4): 697-715.

Pil MW, Baggio RA, Tschá MK, Marteleto FM, Orélis-Riveiro R, Patella L, Chammas M, Ostrensky A, Boeger, WA (2017). The influence of paleoclimate on the distribution on genetic variability and demography of fishes in a large and highly fragmented Neotropical river. *Hydrobiologia*, 805(1): 97–112

Rosa GR, Salvador GN, Bialetzki A, Santos GB (2017). Spatial and temporal distribution of ichthyoplankton during an unusual period of low flow in a tributary of the São Francisco River, Brazil. *River. Res. Appl*., 1–14.
